# Factors associated with duration of breastfeeding in Spain: a cohort study

**DOI:** 10.1186/s13006-020-00324-6

**Published:** 2020-09-09

**Authors:** Carolina Lechosa-Muñiz, María Paz-Zulueta, Sonia Mateo Sota, María Sáez de Adana Herrero, Elsa Cornejo del Rio, Javier Llorca, María J. Cabero-Perez

**Affiliations:** 1 Hospital Universitario Marqués de Valdecilla, 39008 Santander, Spain; 2 Faculty of Nursing, Universidad de Cantabria. IDIVAL, GI Derecho Sanitario y Bioética. GRIDES, 39008 Santander, Spain; 3 Universidad de Cantabria – IDIVAL, Santander, Spain; 4 CIBER Epidemiology and Public Health (CIBERESP), Madrid, Spain

**Keywords:** Breastfeeding, Smoking in pregnancy, Educational level, Maternal age

## Abstract

**Background:**

Factors associated with duration of breastfeeding have been usually studied at specific times after birth. Little is known about how much time is added to breastfeeding by each associated factor.

**Methods:**

A cohort of 969 mother-child dyads was followed-up for twelve months at the Marqués de Valdecilla University Hospital, Spain, in 2018. Data on mother characteristics, pregnancy, delivery and children characteristics were obtained from medical records. Length of breastfeeding was reported by the mothers and recorded in paediatric medical record at hospital discharge and 2, 4, 6, 9 and 12 months of life. Factors associated with duration of breastfeeding were analysed via multivariate Weibull regression parameterized as accelerated time of failure. Results are presented as time ratios.

**Results:**

About four out of five children were breastfed at hospital discharge, although this proportion dropped to 65% in children born from smoker women, 70% in preterm children and 68% in neonates weighting less than 2500 g. Mother’s age was associated with longer breastfeeding, adding 2% more breastfeeding time per year (adjusted time ratio 1.02; 95% confidence interval 1.00, 1.04). Children born from mothers with university studies were breastfed 53% more time than those born from mothers with primary studies (adjusted time ratio 1.53; 95% confidence interval 1.21, 1.95); smoking in pregnancy decreased length of breastfeeding by 41% (adjusted time ratio 0.59; 95% confidence interval 0.46, 0.76). Other factors associated with longer breastfeeding were single pregnancy and newborn weight over 2500 g.

**Conclusions:**

Analysing factors associated with duration of breastfeeding as time parameters allows us to quantify the amount of time gained or lost by each factor, which could make it easier to evaluate the relevance of programmes directed to promote facilitating breastfeeding factors.

## Background

The World Health Organization recommends that “infants should be exclusively breastfeeding for the first six months of life” with further breastfeed supplemented with solid meals until 24 months or more, or for as long as the mother and baby desire [1], and scientific societies in America and Europe adhere this position [2, 3]. Breastfeeding rates reached 56–98% immediately after birth in European countries [4], however, it drops to 38–71% at 6 months after birth and only 13–39% if only exclusively breastfeeding is considered [4].

Factors favouring breastfeeding both onset and continuation, include higher maternal education [5, 6], parity [6, 7], birth at term [5], vaginal delivery [8, 9], maternal smoking [8, 10, 11] and skin-to-skin mother-infant contact shortly after birth [12]. A recent meta-analysis confirmed these results, finding that the influence of these factors on breastfeeding continuation was similar to that of the initiation of breastfeeding and confirmed the importance of previous experience, non-separation of dyads, and education in breastfeeding [13]. The fact that such an influence was measured via relative risk at specific times (e.g. 3 months after birth) put a limitation to its interpretability. For instance, let us consider four women abandoning breastfeeding at 1 week, 2 months + 3 weeks, 3 months + 1 week and 6 months of life, respectively. In such analysis, the first two would have been classified as failure because they were not breastfeeding at 3 months and the other two as success as they were both breastfeeding at 3 months. The second and the third women, however, have much in common with each other regarding the length of breastfeeding than with the others. Instead, we have studied factors associated with breastfeeding continuation using survival techniques. The aim of our study was to estimate not only which factors are related to prolonged breastfeeding, but also the amount of breastfeeding time gained by each factor.

## Methods

### Design and setting

We carried out a prospective cohort study by recruiting 969 consecutive newborns in the University Hospital Marqués de Valdecilla (HUMV), Santander, Spain, from 1st January 2018 to 31st August 2018. The HUMV attends about 3000 deliveries per year and is immersed in the Baby-Friendly Hospital Initiative. Women who rejected to sign the informed consent and those who did live in the region were excluded from the study. Details on recruitment and gathering information have been published elsewhere [11].

### Data collection

Data obtained from maternal medical records included maternal age, parity, educational level, occupational activity, pregnancy duration and type of delivery. Information on smoking in pregnancy was obtained by interview when the mother was admitted for delivery; no information on smoking was obtained in the follow-up. From newborn medical records we gathered her/his gender, weight at birth and whether she/he was singleton or twin. Newborn attendance to child care was obtained by interviewing the mother in each check-up at 2, 4, 6, 9 and 12 months of life.

Type of feeding was obtained at hospital discharge and from the paediatric record of the health checks established in the regional Health Service’s child care program at 2, 4, 6, 9 and 12 months of life. All participants were followed-up for 1 year in order to ascertain the breastfeeding duration.

### Data measures

Educational level was classified as primary studies, secondary studies, short cycle in higher education e.g.: Foundation Degree or similar- and university studies. Occupational activity was classified as working, unemployed, inactive and student. Pregnancy duration was recorded in weeks and days and later categorized as < 34 weeks, 34–36 weeks, ≥ 37 weeks. Type of delivery was grouped in vaginal (non-instrumental), cesarean rate or instrumental. Weight at birth was sorted as low weight (< 2500 g), normal (2500–4000 g) and overweight (> 4000 g).

Type of feeding (exclusive maternal milk, mixed -maternal plus other options, and infant formula milk, defined as in [1]) was obtained at hospital discharge. In this regard, exclusive breastfeeding at discharge is considered for infants who have been exclusively breastfed or who have received expressed breast milk from birth to discharge. In the other time points studied the statistics are obtained with the food received in the last 24 h Thus, exclusively breastfed children are those who have received only breast milk, mixed- children who have received some formula supplement, and infant formula fed. Type of feeding was also obtained from the paediatric record of the health checks established in the regional Health Service’s child care program at 2, 4, 6, 9 and 12 months of life. All participants were followed-up for 1 year in order to ascertain the breastfeeding duration.

### Statistical analysis

The minimum required sample size was *n* = 805. This figure was obtained in order to estimate the proportion of breastfeeding with 5% precision, assuming the worst case scenario (i.e. proportion = 50%) and alpha error = 0.05. Descriptive results are presented as number (percentage) or mean ± standard deviation. Means were compared using ANOVA and percentages via chi-squared test. The analysis was carried out using the time variable was length of breastfeeding and participants interrupting breastfeeding were considered events. Estimates were obtained using the Kaplan-Meier method. The relationships between woman and newborn characteristics and length of breastfeeding were analysed via Weibull regression parameterized as accelerated time of failure [14]. Of note, Weibull regression could be parameterized in two ways, proportional hazards or accelerated time of failure. The most frequent parameterization is proportional hazards, then, the main result is usually expressed as hazard ratio. Accelerated time of failure parameterization, however, displays its main result as time ratio, as time is a more natural and immediately understandable unit than hazard, time ratios are easier to interpret. For instance, time ratio = 2 would mean length of breastfeeding doubled that of the reference category and time ratio = 0.5 would mean length of breastfeeding halved that of the reference category. After the Weibull analysis took place, we estimated adjusted medians and means of breastfeeding duration according to different mother or child characteristics.

## Results

**Fig. 1:**
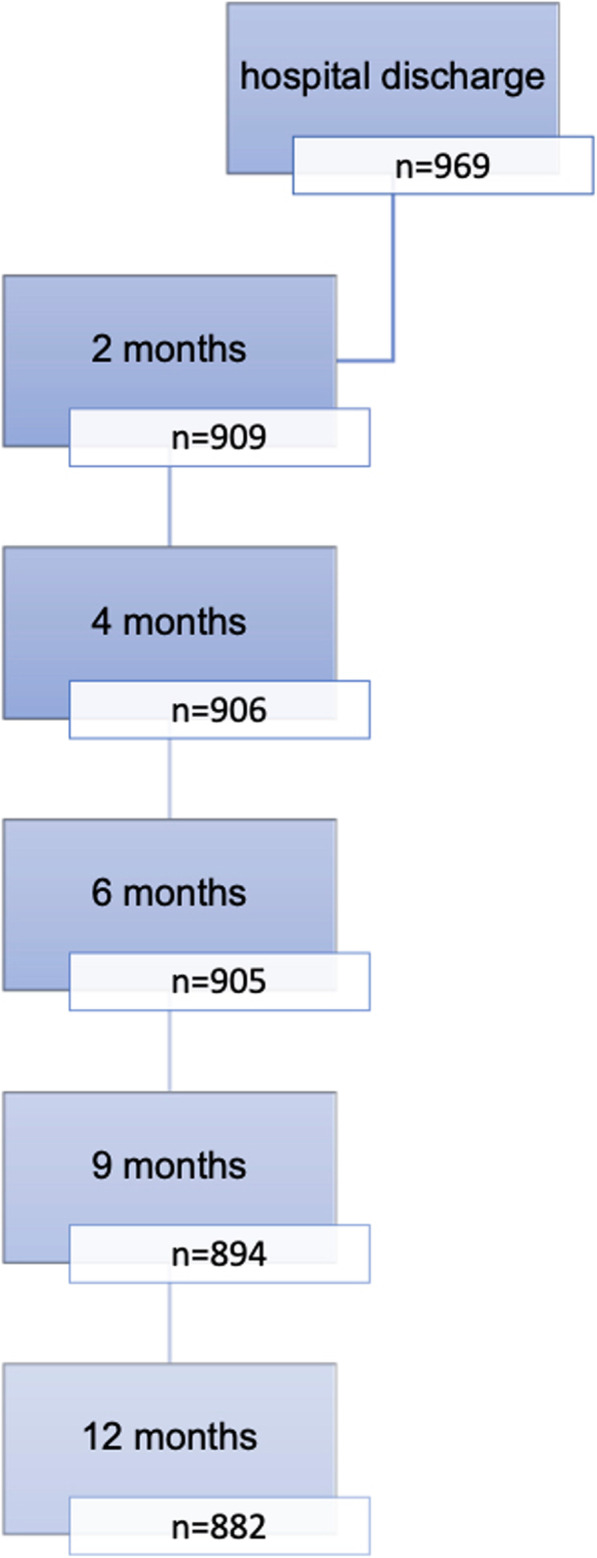
Flowchart

The initial sample was 992 infants included in the study at birth. A child was excluded because she died at 2 months of age, she was born in the 25th week of gestation, weighting 870 g, she was immediately admitted in the pediatric ICU and had never the opportunity of being breastfed. Twenty-two children were excluded because their parents were not residing in the region. Finally, 969 newborns from 949 pregnancies were included in the analysis, and 882 children (91%) were followed-up until 12 months. A flowchart with the recruitment and follow-up data appears in Fig. 1.

Mothers were 33.7 ± 5.2 years on average; more than one in three had attended university studies (*n* = 350, 36.9%) and about 70% were active workers. One in eight women declared to have smoked in pregnancy.

Only 59 (6.1%) newborns were preterm and 40 (4.1%) were twins.

More than 90% newborns weighted between 2500 and 4000 g at birth, 83 (8.6%) weighted less than 2500 g and 78 (8.1%) weighted more than 4000 g. More than 50% newborns were breastfed at hospital discharge (Table 1).

**Table 1:** Main characteristics of participants in the study

Variable	Category	All participants (***N*** = 969)	Participants with exclusive breastfeeding at hospital discharge (***N*** = 520)	Participants without exclusive breastfeeding at hospital discharge (***N*** = 449)	***p*** value
Maternal age	Mean ± sd	33.7 ± 5.2	33.7 ± 5.0	33.6 ± 3.9	0.76*
Maternal educational level	Primary studies	215 (22.7)	91 (17.6)	124 (27.6)	< 0.001**
	Secondary studies	112 (11.8)	60 (11.6)	53 (11.8)	
	Foundation degree	272 (28.7)	147 (28.4)	134 (29.8)	
	University studies	350 (36.9)	220 (42.5)	138 (30.7)	
Maternal occupation	Working	660 (69.6)	368 (71.0)	303 (67.5)	0.64**
	Unemployed	163 (17.2)	84 (16.2)	85 (18.9)	
	Inactive	116 (12.2)	61 (11.8)	56 (12.5)	
	Student	10 (1.1)	5 (1.0)	5 (1.1)	
Smoking in pregnancy	No	830 (87.5)	467 (90.2)	378 (84.2)	0.005**
	Yes	119 (12.5)	51 (9.8)	71 (15.8)	
Pregnancy duration	≥ 37 weeks	897 (94.5)	501 (96.7)	408 (90.9)	0.001**
	34–36 weeks	36 (3.8)	11 (2.1)	28 (6.2)	
	< 34 weeks	16 (1.7)	6 (1.2)	13 (2.9)	
Type of delivery	Vaginal (non-instrumental)	653 (67.4)	391 (75.2)	262 (58.4)	< 0.001**
	Instrumental	80 (8.3)	43 (8.3)	37 (12.9)	
	Cesarean rate	236 (24.4)	86 (16.5)	150 (33.4)	
Newborn gender	Male	490 (50.6)	255 (49.0)	235 (52.3)	0.31**
	Female	479 (49.4)	265 (51.0)	214 (47.7)	
Twin pregnancy	No	929 (95.9)	516 (99.2)	413 (92.0)	< 0.001**
	Yes	40 (4.1)	4 (0.8)	36 (8.0)	
Newborn weight	< 2500	83 (8.6)	23 (4.4)	60 (13.4)	< 0.001**
	2500–4000 g	808 (93.4)	463 (89.0)	345 (76.8)	
	> 4000 g	78 (8.1)	34 (6.5)	44 (9.8)	
Breastfeeding duration	Mean ± sd	5.9 ± 5.2	7.8 ± 4.6	3.9 ± 4.9	< 0.001*
	Yes	520 (53.7)	520 (100.0)	–	
Attendance to child care	No	763 (78.7)	398 (76.5)	365 (81.3)	0.09**
	Yes	132 (13.6)	74 (14.2)	58 (12.9)	
	Unknown	74 (7.6)	48 (9.2)	26 (5.8)	

*ANOVA. **Chi-squared test

**Fig. 2:**
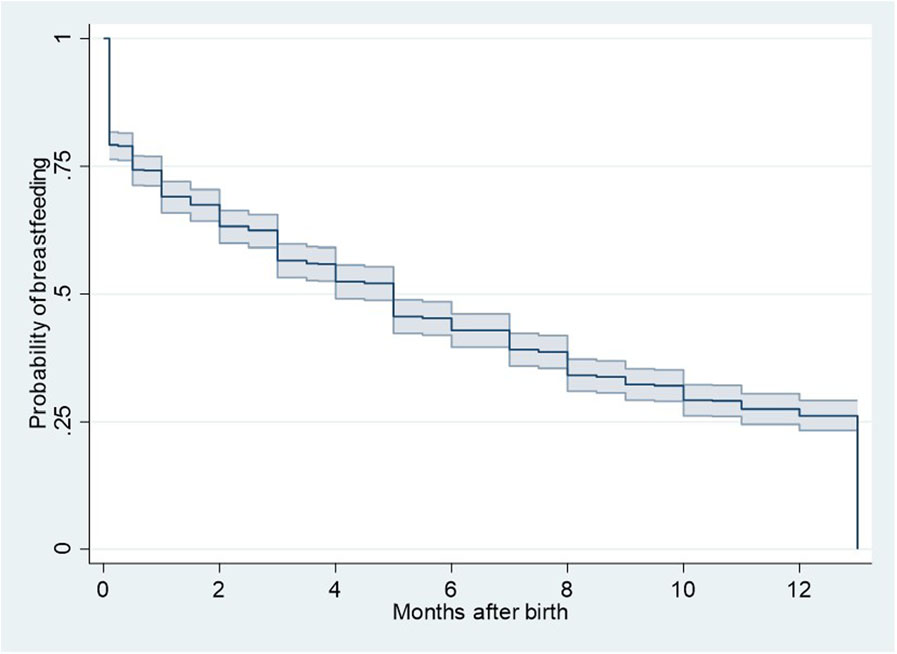
Duration of breastfeeding in the whole cohort. Kaplan-Meier estimates with 95% confidence bands

**Fig. 3:**
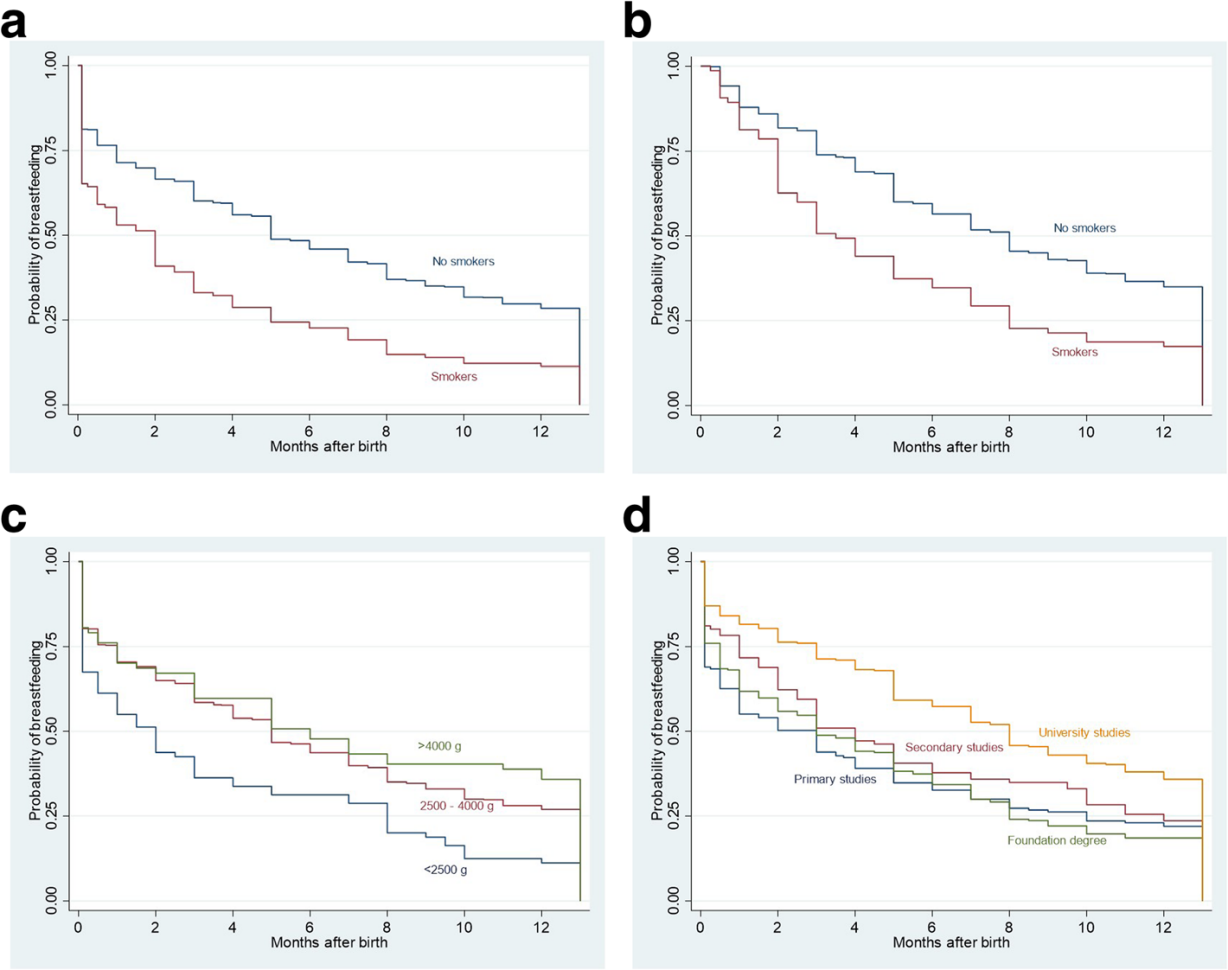
Duration of breastfeeding according to (**a**) maternal smoking in the whole cohort, (**b**) maternal smoking only if children were breastfed at hospital discharge, (**c**) newborn weight and (**d**) maternal occupation

About 79% children were breastfed at hospital children, and the percentage dropped to 57, 43 and 32% at 3, 6 and 9 months after birth, respectively (Table 2 and Fig. 2). Children born from smoker women were less likely breastfed than those born from non-smoker women (65% vs. 81% at hospital discharge and 23% vs. 46% at 6 months) (Table 2 and Fig. 3a); these differences still hold when analysing only children with exclusive maternal milk at hospital discharge (Fig. 3b). About 80% children born at term were breastfed, contrasting with less than 70% of preterm children, and those with gestation shorter than 34 weeks had a faster decline in breastfeeding (Table 2). Newborn weight lower than 2500 g was associated with lower percentages of breastfeeding than newborn weight between 2500 and 4000 g, 68% vs 80% at hospital discharge and 31% vs 44% at 6 months (Table 2 and Fig. 3c). Children born from women with university studies were more likely to be breastfed than those born from women with lower educational level (Table 2 and Fig. 3d).

**Table 2:** Percentage of newborns breastfed at birth, 3, 6 and 9 months: according to different maternal or newborn factors

Factor	At hospital discharge	3 months	6 months	9 months
All participants	79.2 (76.4, 81.7)	56.6 (53.2, 59.8)	42.9 (39.6, 46.1)	32.2 (29.2, 35.3)
Maternal educational level				
Primary studies	69.0 (61.8, 75.1)	43.9 (36.7, 50.8)	32.6 (26.0, 39.4)	26.2 (20.1, 32.7)
Secondary studies	81.1 (72.3, 87.4)	50.9 (41.1, 60.0)	37.7 (28.6, 46.9)	34.9 (26.0, 44.0)
Foundation degree	76.0 (70.2, 80.8)	48.8 (42.5, 54.8)	34.3 (28.5, 40.1)	22.1 (17.2, 27.3)
University studies	86.9 (82.7, 90.2)	71.3 (66.1, 76.0)	57.3 (51.7, 62.5)	43.0 (37.5, 48.3)
Maternal occupation				
Working	79.2 (75.7, 82.2)	59.2 (55.2, 63.0)	43.4 (39.5, 47.3)	31.5 (27.8, 35.2)
Unemployed	59.2 (55.2, 63.0)	53.6 (45.4, 61.2)	43.7 (35.7, 51.4)	35.8 (28.2, 43.4)
Inactive	78.1 (68.9, 84.9)	44.8 (35.1, 54.0)	37.1 (28.0, 46.3)	31.4 (22.8, 40.4)
Student	100	66.7 (28.2, 87.8)	55.6 (20.4, 80.5)	33.3 (7.8, 62.3)
Smoking in pregnancy				
No	81.3 (78.4, 83.9)	60.1 (56.6, 63.5)	45.9 (42.3, 49.4)	35.0 (31.6, 38.4)
Yes	65.2 (55.8, 73.1)	33.0 (24.6, 41.7)	22.6 (15.5, 30.6)	13.9 (8.3, 20.9)
Duration of pregnancy				
≥ 37 weeks	80.1 (77.2, 82.7)	57.5 (54.0, 60.8)	43.6 (40.2, 47.0)	33.0 (29.8, 36.2)
34–36 weeks	65.8 (48.5, 78.5)	50.0 (33.4, 64.5)	34.2 (19.8, 49.1)	26.3 (13.7, 40.8)
< 34 weeks	68.4 (42.8, 84.4)	31.6 (12.9, 52.3)	26.3 (9.6, 46.8)	10.5 (1.8, 28.4)
Type of delivery				
Vaginal (non-instumental)	80.6 (77.1, 83.6)	57.8 (53.7, 61.6)	43.8 (39.7, 47.8)	33.1 (29.3, 37.0)
Instrumental	80.0 (69.0, 87.4)	58.7 (46.7, 68.8)	40.0 (28.9, 50.8)	26.7 (17.3, 37.0)
Cesarean rate	75.1 (68.7, 80.4)	52.6 (45.7, 59.0)	41.3 (34.7, 47.8)	31.9 (25.8, 38.2)
Newborn gender				
Male	79.0 (74.9, 82.6)	58.8 (54.0, 63.2)	44.9 (40.2, 49.5)	34.4 (30.0, 38.9)
Female	79.4 (75.3, 82.9)	54.4 (49.6, 58.9)	40.8 (36.2, 45.4)	30.1 (25.8, 34.4)
Twin pregnancy				
No	79.9 (77.0, 82.5)	57.3 (53.9, 60.6)	43.7 (40.3, 47.0)	33.0 (29.9, 36.2)
Yes	64.1 (47.0, 76.9)	41.0 (25.7, 55.8)	25.6 (13.3, 39.9)	15.4 (6.2, 28.3)
Newborn weight				
< 2500	67.5 (56.1, 76.6)	36.3 (25.9, 46.7)	31.3 (21.5, 41.5)	18.8 (11.1, 28.0)
2500–4000 g	80.4 (77.3, 83.1)	58.5 (54.9, 62.0)	43.7 (40.1, 47.3)	33.0 (29.6, 36.4)
> 4000 g	80.6 (68.9, 88.2)	59, 7 (47.0, 70.3)	47.8 (35.5, 59.1)	40.3 (28.6, 51.7)

Note. Kaplan-Meier estimates and 95% confidence intervals

Table 3 displays results from Weibull regression. Each additional year in maternal age increased breastfeeding time by 2% in the multivariate analysis (adjusted time ratio 1.02; 95% confidence interval (CI) 1.00, 1.04). Women with university studies breastfed for 53% more time than women with primary studies (adjusted time ratio 1.53; 95% CI 1.21, 1.95). Women who smoked in pregnancy almost halved the time of breastfeeding (adjusted time ratio 0.59; 95% CI 0.46, 0.76) as also did twin pregnancy (adjusted time ratio 0.57; 95% CI 0.37, 0.89). Newborns weighting less than 2500 g were breastfed less time than newborns with normal weight, although this result was not statistically significant in the multivariate analysis (adjusted time ratio 0.77; 95% CI 0.53, 1.11).

**Table 3:** - Factors associated with breastfeeding duration: results obtained by Weibull regression

Factor	Time ratio (95% CI)	***p***	Adjusted time ratio (95% CI)^a^	***p***
Maternal age (per year)	1.03 (1.01, 1.04)	0.002	1.02 (1.00, 1.04)	0.03
Maternal educational level				
Primary studies (ref.)	1	–	1	–
Secondary studies	1.26 (0.93, 1.70)	0.14	1.28 (0.95, 1.74)	0.11
Foundation degree	1.05 (0.83, 1.34)	0.68	0.99 (0.77, 1.26)	0.92
University studies	1.73 (1.37, 2.17)	< 0.001	1.53 (1.21, 1.95)	< 0.001
Maternal occupation				
Working (ref.)	1	–		
Unemployed	0.99 (0.79, 1.24)	0.93		
Inactive	0.84 (0.64, 1.09)	0.19		
Student	1.20 (0.51, 2.79)	0.68		
Smoking in pregnancy				
No (ref.)	1	–	1	–
Yes	0.51 (0.40, 0.66)	< 0.001	0.59 (0.46, 0.76)	< 0.001
Duration of pregnancy				
≥ 37 weeks (ref.)	1	–	1	–
34–36 weeks	0.75 (0.49, 1.13)	0.17	0.97 (0.62, 1.53)	0.91
< 34 weeks	0.55 (0.30, 0.98)	0.04	0.91 (0.46, 1.79)	0.78
Type of delivery				
Vaginal (non-instrumental)(ref.)	1	–		
Instrumental	0.93 (0.68–1.27)	0.66		
Cesarean rate	0.93 (0.76–1.13)	0.46		
Newborn gender				
Male (ref.)	1	–		
Female	0.92 (0.78, 1.10)	0.37		
Twin pregnancy				
No (ref.)	1	–	1	–
Yes	0.56 (0.37, 0.85)	0.006	0.57 (0.37, 0.89)	0.01
Newborn weight				
< 2500	0.62 (0.46, 0.83)	0.001	0.77 (0.53, 1.11)	0.16
2500–4000 g	1	–	1	–
> 4000 g	1.11 (0.81, 1.53)	0.52	1.08 (0.79, 1.48)	0.63

^a^Time ratio adjusted for the remaining variables in the table. Occupation, type of delivery and gender were excluded from the multivariate analysis because of their lack of relationship with breastfeeding duration in the crude analysis

Note: that results are not express as hazard ratios but as time ratios. Therefore, values higher than 1 indicate longer breastfeeding, while values lower than 1 indicate the opposite. For instance, time ratio = 0.55 in participants with pregnancy duration lower than 34 weeks means that breastfeeding duration in those participants about halved that of participants with pregnancy duration > 37 weeks

In order to better explain the relevance of multivariate results in Table 3, let us suppose we want to compare two groups of mothers: group A are mothers 35 years old, with university studies and smokers and group B are mothers 30 years old, with primary studies and no smokers. Then, using results from the multivariate model in Table 3, time ratio (TR) comparing group A with group B can be estimated as:

$$ TR={TR}_{age}^{35-30}\times {TR}_{university}\times {TR}_{smoking}={1.02}^5\times 1.53\times 0.59=1.00, $$

showing that the deleterious effect of smoking in pregnancy cancels out the combined positive effects of five additional years and university studies on breastfeeding duration.

To further clarify the impact each variable included in Table 3 multivariate model had on breastfeeding duration, Table 4 displays adjusted means and medians of breastfeeding duration. All means were higher than medians, indicating the distribution of breastfeeding duration is right skewed. Average duration of breastfeeding was 0.98 months longer for women aged 35 than for those aged 20; 2.24 months longer for women with university studies than for those with primary studies and 2.06 shorter for smokers than for non-smokers.

**Table 4:** Breastfeeding duration in months

Factor	Mean (95% CI)	Median (95% CI)
Maternal age (selected ages)		
20 years	5.05 (4.09–6.00)	2.63 (1.97–3.29)
25 years	5.37 (4.72–6.03)	2.90 (2.38–3.41)
30 years	5.70 (5.29–6.11)	3.18 (2.80–3.56)
35 years	6.03 (5.69–6.36)	3.50 (3.15–3.85)
Maternal educational level		
Primary studies (ref.)	5.03 (4.30, 5.77)	2.78 (2.22, 3.33)
Secondary studies	5.82 (4.87, 6.76)	3.57 (2.67, 4.46)
Foundation degree	4.95 (4.34, 5.56)	2.74 (2.29, 3.20)
University studies	7.27 (6.72, 7.81)	4.28 (3.66, 4.90)
Smoking in pregnancy		
No (ref.)	6.21 (5.86, 6.56)	3.62 (3.25, 3.99)
Yes	4.15 (3.23, 5.08)	2.14 (1.61, 2.66)
Duration of pregnancy		
≥ 37 weeks (ref.)	5.97 (5.62, 6.31)	3.46 (3.10, 3.81)
34–36 weeks	5.91 (4.26, 7.56)	3.37 (1.88, 4.85)
< 34 weeks	4.96 (2.38, 7.54)	3.13 (1.01, 5.25)
Twin pregnancy		
No (ref.)	6.02 (5.69, 6.36)	3.51 (3.15, 3.87)
Yes	4.16 (2.51, 5.80)	2.00 (1.13, 2.87)
Newborn weight		
< 2500 g	5.01 (3.71, 6.30)	2.68 (1.74, 3.63)
2500, 4000 g	6.00 (5.63, 6.36)	3.49 (3.11, 3.86)
> 4000 g	6.49 (5.30, 7.67)	3.77 (2.61, 4.92)

Note: Medians and means adjusted for the maternal age and the remaining variables in the table. Only variables in the multivariate analysis of Table 3 are included

## Discussion

In this cohort study, we have analysed how length of breastfeeding is conditioned by maternal characteristics (age, education, occupation activity, smoking habit), pregnancy features (duration, twin/single, type of delivery) and newborn attributes (gender, weight at birth). In a multivariate analysis that provides length of breastfeeding ratios, we found that breastfeeding was shorter in children born from mothers who were younger, without university studies or smoked in pregnancy, and those born from twin pregnancy or weighting less than 2500 g at birth.

The lack of quality information in previous studies prevented Cohen et al. from including maternal age in their meta-analysis on factors associated with breastfeeding initiation and continuation [13]. According to our results, each additional year in maternal age increases breastfeeding duration by 2% (i.e. time ratio = 1.02); in this regard, if women aged 25 are expected to breastfed 2.90 months on median, women 10 years older are expected to breastfed about 20% more time, until 3.50 months on median (Table 4). Scott et al. [15] found that 10 years more doubled the odds of breastfed continuation, although they did not study breastfeeding duration but its continuation until 4 months.

To study maternal education as factor associated with breastfeeding continuation is not straight forward as education attainment could be measured in different ways as educative systems are not always equivalent to each other. The easiest ways of making international results comparable are (i) measuring it in number of years of schooling [15], which assumes a linear effect (i.e. each additional educative year has the same effect on breastfeeding length) and (ii) restricting the analysis to a comparison between the highest and the lowest educational levels [13]. Both strategies mislay part of the information whether assuming a linear effect or omitting intermediate educational levels. We have found that, far from linear, the effect of maternal education in enlengthening breastfeeding only appears in mothers who have university studies and their children were breastfed for 53% more time than those born from mothers with only primary studies. Of note, in the last decades, progressively higher proportions of Spanish women have attained university degrees, making it possible for 37% women in our cohort and even a higher percentage among those breastfeeding at hospital discharge (Table 1). Mechanisms for higher educated women to breastfeed longer are not clear. It has been suggested that they are more aware of the health implications of breastfeeding [16] and that they -being economically more independent than less educated women, and are empowered to make the decision on whether breastfeeding or not by themselves [17].

Smoking in pregnancy has been largely and consistently identified as factor associated with both breastfeeding non-initiation and early discontinuation [10, 11, 13]. What our study adds is that breastfeeding lasted about 40% less in children born from mothers who smoked in pregnancy when compared with those from mothers who did not smoke. Among the identified factors influencing breastfeeding initiation and duration, smoking is probably the more modifiable and the more consistently associated with other deleterious effects on both mothers and children [18–20]. In spite of that, about 1 in 8 women in our cohort smoked in pregnancy, a similar percentage as reported in the US [13].

Shorter duration of pregnancy and lower weight at birth have been frequently found related to early breastfeeding discontinuation [8, 9, 21]. The fact that these two factors are strongly associated with each other makes it difficult to separate its effects. According to our results, the association between length of pregnancy and breastfeeding duration disappeared in the multivariate setting when adjusting for weight at birth, suggesting that lower weight at birth is the dominant factor of this finding.

The main point our study adds to literature is to present results as time ratios instead of the usual hazard ratios. Time ratios allows an easier interpretation, especially in the multivariate model where we can take advantage of its multiplicative nature. In this regard, we have shown an example on how a deleterious factor (smoking) could cancel out some positive factors (education level and maternal age), which reinforces the importance of acting on any preventable factor, whatever the exposure to the non-modifiable factors is.

Our study has some limitations. Firstly, length of breastfeeding was reported by mothers, so there is some room for information bias as some women could have informed according to social desires more than according to their actual practice. Secondly, although our sample size is close to 1000 mother-child dyads, some categories in the analysis have few participants, which makes some confidence intervals excessively wide; this could be the case of newborn weight lower than 2500 g or some categories in duration of pregnancy in the multivariate setting. Third, we have limited our research to variables standardly recorded in order to make our results more robust, but this strategy has left aside some important variables related to breastfeeding initiation and continuation, such as breastfeeding self-efficacy, return to work activity or previous breastfeeding experience. The main strength of our study is that women and children have been prospectively followed in a homogeneous way in a single centre committed to breastfeeding practices.

## Conclusion

Using data from a prospective cohort study we have demonstrated the impact of several maternal and child factors on breastfeeding duration. The current trends to higher maternal age and increasing percentage of women with university studies would favour longer breastfeeding, on the other hand, a relatively high percentage of women smoking in pregnancy and a trend to increasing preterm births would counterbalance those benefits.

## Data Availability

Data cannot be made publicly available in order to protect patient privacy. The data are available on request from the University of Cantabria Archive (http://repositorio.unican.es/) for researchers who meet the criteria for access to confidential data. Requests may be sent to the Ethics Committee (ceicc@idival.org) or Dr. María Paz-Zulueta (maria.paz@unican.es).

## Abbreviations

CI: Confidence interval
HUMV: Hospital Universitario Marqués de Valdecilla
ICU: Intensive Care Unit
SD: Standard deviation
